# Modeling of growth performance, physiological response, and intestinal microbiota shift in growing Japanese quail fed olive leaf powder

**DOI:** 10.3389/fvets.2026.1824857

**Published:** 2026-05-22

**Authors:** Saad A. Al-Ardhi, Saad K. Al-Waeli, Moustafa Amin Osman, Eman A. Al-Shahari, Nawal H. Siddig, Ahmed Ezzat Ahmed, Yehia Hazzazi, Mari Sumayli, Mohammed Al-Rasheed, Ibrahim Mufadhi M. Alanazi

**Affiliations:** 1 Department of Animal Production, College of Agriculture, Al-Muthanna University, Al-Muthanna, Iraq; 2 Regional Centre for Food and Feed, Agricultural Research Centre, Ministry of Agriculture and Land Reclamation, Giza, Egypt; 3 Biology Health Specialties, Basic Sciences and Their Applications Unit, Applied College at Muhayil Asir, King Khalid University, Abha, Saudi Arabia; 4 Department of Mathematical Science, College of Science, Princess Nourah bint Abdulrahman University, Riyadh, Saudi Arabia; 5 Biology Department, College of Science, King Khalid University, Abha, Saudi Arabia; 6 Department of Biology, College of Science, Jazan University, Jazan, Saudi Arabia; 7 Department of Clinical Sciences, College of Veterinary Medicine, King Faisal University, Al-Ahsa, Saudi Arabia; 8 Department of Pharmacology and Toxicology, Faculty of Medicine, Umm Al-Qura University, Makkah, Saudi Arabia

**Keywords:** blood indicators, cecal microbiota, growth performance, olive leaf powder, quail

## Abstract

**Introduction:**

Due to the anti-inflammatory, antioxidant, and antibacterial properties of olive leaf powder (OLP), it may serve as a beneficial feed supplement for birds. This study aimed to evaluate the effect of adding OLP to feed on growth efficiency, carcass traits, blood parameters, antioxidant activity, and cecal microbial load in growing Japanese quail.

**Methods:**

Three hundred and seventy-five one-day-old quail chicks were randomly assigned to five experimental groups, each containing five replicates of 15 birds: a control group fed a basal feed and four experimental groups given 3, 4, 5, and 6% OLP per kg of quail diets for the 6 weeks.

**Results:**

The results showed a significant (*P* < 0.05) improvement in growth performance, with increased live body weight (LBW) and an insignificantly improved feed conversion ratio in the 6% OLP group. Carcass yield and total edible meat also substantially improved (*P* < 0.05). Blood parameters showed improved (*P* < 0.05) protein and lipid levels and increased liver enzyme activity; moreover, the use of the OLP led to a decrease in TC, TG, LDL, and VLDL levels, as well as an increase in HDL. Additionally, the activity of antioxidant enzymes increased (*P* < 0.001), along with higher levels of GSH and SOD activity. All meat quality attributes, including cooking loss %, water-holding capacity, PH values, and meat color, improved (*P* < 0.05) with the use of OLP. Furthermore, intestinal microbiota analysis showed a significant improvement in *Lactobacillus* count and a decrease in pathogenic bacteria (total bacterial count, coliforms, *E. coli*, and *Salmonella*). However, the *Bacillus* count was not significantly affected by OLP supplementation.

**Discussion:**

The findings of this study demonstrate that OLP is a promising natural feed additive for growing Japanese quail, enhancing growth performance and the gut microbiome, thereby establishing it as a safe and natural growth promoter.

## 1 Introduction

The steady population growth has led to an increasing demand for animal protein, necessitating plans to increase production in efficient and economic ways (1, 2). Poultry meat, particularly quail meat, is a major source of protein due to its low cost, rapid production, and nutritional quality (3). However, the poultry industry faces challenges in producing antibiotic-free and environmentally safe meat, leading to a search for natural feed additives that enhance productivity and reduce disease and mortality (4, 5). As a result, replacing antibiotics used as growth promoters with effective natural alternatives, such as plant products and herbal extracts, is a global trend (6, 7).

In this context, natural additives and their extracts are rich in active ingredients that act as antibacterial and growth promoters, providing nutritional and health benefits for poultry (8, 9). For example, Olive trees are characterized by their high content of natural antioxidants such as oleuropein, dimethyloleuropein, ligstroside, and oleoside, as well as phenolic compounds such as tyrosol, caffeic acid, tocopherols, catechol, rutin, and hydroxytyrosol, in addition to flavonoids such as kaempferol, apigenin, and luteolin; all these compounds possess significant medicinal potential (10).

Beyond growth promotion, OLP is a natural alternative to antibiotics for bacterial infections in poultry, especially *Salmonella*. Its bioactive compounds, such as oleuropein and hydroxytyrosol, work by breaking down bacterial cell membranes, stopping biofilm formation, and slowing bacterial growth (11). Thanks to its potent antimicrobial, anticoccidial, antioxidant, and anti-inflammatory properties (12), olive leaf extract shows promise in improving poultry health and reducing the risk of *Salmonella enterica* contamination of eggs and meat.

Previous research has demonstrated the potential benefits of olive leaf products in poultry diets. Adding olive leaf extract (OLE) to quail diets resulted in increased body weight, improved feed conversion ratio, decreased MDA levels, and enhanced glutathione reductase and SOD activity in both the liver and heart (13). Furthermore, Bahshwan et al. (14) observed an improvement in intestinal microbial load, characterized by an increase in lactic acid bacteria and a decrease in Salmonella and *E. coli*, when olive leaf extract was added to the diets of Japanese quail. Similarly, a significant decrease in HDL levels and *E. coli* count in the cecum was also observed in broiler chickens when olive leaf extract was added to their diets at varying ratios (15). Furthermore, the addition of 150 mg/kg of OLE enhanced the lipid levels, growth performance characteristics, antioxidant activity, and immunity of laying hens (16).

Previous research has shown that olive OLP can be good for poultry, but there are still some important gaps in our knowledge. Although OLP holds potential as growth promoters and alternative feed additives in poultry nutrition, further investigation is necessary to fully clarify their efficacy, mechanisms of action, and antimicrobial properties. Most of the research that has been done so far has been on broiler chickens or laying hens, and there is not much data on growing Japanese quail. Moreover, most studies have utilized olive leaf extract instead of the more cost-effective powdered form, and dose–response relationships across a broad spectrum of inclusion levels (from 3 to 6%) have not been systematically assessed in this species. Furthermore, the simultaneous evaluation of growth performance, blood biochemistry, antioxidant status, meat quality, and cecal microbiota within a single experimental model is still limited. This study aimed to investigate the effects of four different doses of OLP on growth, hematological parameters, antioxidant levels, immune response, and gut microbiota composition in Japanese quail, thereby offering a thorough assessment of OLP as a potential natural feed additive for this species.

## 2 Materials and methods

### 2.1 Ethical approval

This study was performed on a private poultry farm in the Sharqia Governorate and at the Animal Production Research Institute laboratory in Dokki, Giza, Egypt, with ethical approval from the Institute’s Research Ethics Committee, reference number IACUC-2023. The committee approved all experimental procedures, including the breeding and management of birds.

### 2.2 Preparation of olive leaf powder (OLP) and extract (OLE)

The study utilized olive leaves acquired from a commercial source. For chemical analysis, employ reagents such as gallic acid, quercetin, methanol, aluminum chloride, foline, ethanol, and DPPH (2,2-diphenyl-1-picrylhydrazyl) sourced from Merck, Darmstadt, Germany.

Olive leaves were first washed with distilled water and air-dried, then oven-dried at 40–45 °C until weight stabilized. The dried leaves were ground into a fine powder (OLP) using a laboratory mill and stored in airtight containers at room temperature until incorporation into the quail diets at levels of 3, 4, 5, and 6%.

For the sole purpose of determining total phenolic (TP) and total flavonoid (TF) content and antioxidant activity, a methanolic extract was prepared. Exactly 10 g of OLP were mixed with 200 mL of 70% methanol (v/v) in a 500 mL Erlenmeyer flask, yielding a solvent-to-sample ratio of 20:1 (mL/g). The mixture was stirred continuously using a magnetic stirrer at 500 rpm for 3 h at room temperature (25 ± 2 °C) in the dark to prevent photodegradation of sensitive phenolic compounds. After extraction, the mixture was filtered through Whatman No. 1 filter paper under vacuum. The filtrate was collected, and the methanol was removed under reduced pressure using a rotary evaporator (Büchi Rotavapor R-300, Flawil, Switzerland) at 45 °C and 100 mbar pressure. The dried extract (OLE) was weighed to calculate extraction yield, then transferred into amber glass vials, flushed with nitrogen gas to prevent oxidation, and stored at −20 °C until analysis (17, 18).

According to Altıok et al. (19), the TP components were analyzed by HPLC, while total TF content was estimated using a method by Ordonez et al. (20). Furthermore, antioxidant activity was measured through the DPPH assay (20). Table 1 presents the TP content (mg GAE g^−1^ extract), TF content (mg QE g^−1^ extract), and DPPH activity (SC_50_; mg mL^−1^) of the methanolic extract (OLE) derived from olive leaves.

**Table 1:** Total phenols, flavonoids, and antioxidant activity of the methanolic extract acquired from olive leaves.

Items	Content
TP (mg GAE g^−1^ extract)	147.28
TF (mg QE g^−1^ extract)	41.70
DPPH activity (SC_50_; mg mL^−1^)	60.89

TP, total phenol; TF, total flavonoid; DPPH, 2,2-diphenyl-1-picrylhydrazyl.

### 2.3 Birds, management, and design

Three hundred and seventy-five one-day-old, unsexed Japanese quail chicks with a similar average initial live weight were randomly distributed into five groups of 75 chicks each, with five replicates of 15 birds each; Unsexed chicks were used to reflect standard commercial production practices, as sexing at hatch is not routinely performed due to the lack of easily distinguishable sexual characteristics until 2–3 weeks of age. The basal diet was formulated according to the recommendations of the National Research Council (21). Table 2 shows the components and chemical composition of the basal diet. The quail chicks in the control group were fed the basal diet without additives, while the diets of the other four groups (OLP1-OLP4) were supplemented with OLP at ratios of 3, 4, 5, and 6%, respectively, for six weeks. During the experiment, the birds were reared in electrically heated battery cages with a constant supply of water and feed, and a continuous lighting program was implemented.

**Table 2:** Basal diet composition and calculated analysis.

Ingredients	%
Maize corn	53.50
Soybean meal (44%)	30.50
Corn gluten meal (60%)	9.50
Wheat bran	1.50
Vegetable oil	0.50
DL-methionine	0.20
L-Lysine HCl	0.30
NaCl	0.50
Limestone	1.0
Mineral premix and vitamins^a^	0.50
Di calcium phosphate (DCP)	2.0
Calculated analysis	
Metabolizable energy (kcal/kg)	2900.0
Crude protein %	24.00
Crude fiber %	3.60
Ca %	1.25
Available phosphorus %	0.40
Lysine	1.36
Methionine	0.63
Methionine + Cystine	0.88

a Premix provided per kg of diet: vitamin A, 12.000 IU; vitamin D3, 2.400 IU; vitamin E, 30 mg; vitamin K3, 4 mg; vitamin B1, 3 mg; vitamin B2, 7 mg; vitamin B6, 5 mg; vitamin B12, 15 μg; niacin, 25 mg, Fe, 80 mg; folic acid, 1 mg; pantothenic acid, 10 mg; biotin, 45 mg; choline, 125,000 mg; Cu, 5 mg; Mn, 80 mg; Zn, 60 mg; Se, 150 μg.

### 2.4 Data collection

All birds were individually weighed at 0, 2, 4, and 6 weeks of age to record LBW. Body weight gain (BWG) was calculated as the difference between the final and initial body weight over each period. Daily feed intake (DFI) was calculated by subtracting the remaining feed from the amount dispensed. The feed conversion ratio (FCR) was calculated as DFI (g) / BWG (g). Both BWG, FI, and FCR were calculated on a cumulative basis over defined experimental periods (1–15, 16–28, 29–42 days, and overall, 1–42 days).

At the end of the experiment, five birds from each group were isolated and kept without feed overnight, with free access to water. The birds were then weighed, slaughtered according to traditional Islamic methods by cutting the jugular vein, and bled for approximately 2–3 min. All carcass characteristics were calculated as a percentage of LBW (live body weight). Blood samples were also collected from each bird during the bleeding process.

### 2.5 Blood indicators

Two blood samples were collected from each slaughtered bird. The first sample was collected in a heparin-free tube, and the serum was centrifuged at 3000 rpm for 15 min. The serum was kept at −20 °C until its biochemical properties were measured. The activity of the liver enzymes alanine aminotransferase (ALT) and aspartate aminotransferase (AST) in the serum was measured using calorimetry. Total protein (TP), albumin (ALB), globulin (GLO), triglycerides (TG), total cholesterol (TC), high-density lipoprotein (HDL), and low-density lipoprotein (LDL) levels were estimated according to the manufacturer’s instructions (22, 23). Oxidation status parameters, including reduced glutathione (GSH), malondialdehyde (MDA), and superoxide dismutase (SOD), were determined using standard biochemical methods.

The second blood specimen was collected in a tube containing heparin and stored at 4 °C until blood parameters were checked. Using a Neubauer hemocytometer improved Natt & Herrick’s solution as a diluent, allowing for the counting of white blood cells (24).

### 2.6 Meat quality

Three specimens of chicken breast fillet were prepared and cut into small pieces (3 cm). The color indices of the meat samples were assessed as follows: brightness (*L*), redness (*a*), and yellowness (*b*) (25). The pH of the breast meat samples was measured using a HANNA pH meter (Won Sookit, USA). Approximately 10 g of the muscle samples were cooked in an aluminum pan placed in an electric oven (preheated to 200 °C) for 15 min or until the oven temperature reached approximately 80 °C. The percentage of weight loss during cooking was estimated by assessing the weight differences between the cooked and raw meat samples, expressed as a percentage of the uncooked beef samples after a 30-min cooling period at 15 °C. Water retention capacity (WHC) was assessed according to the methodology described by Hoff-Lonergan and Lonergan (26).

### 2.7 Cecal microbial load

The total bacterial count (TBC), along with the counts of coliforms, *Lactobacillus*, *Salmonella*, and *E. coli*, was determined using ten grams of ileum samples obtained from the birds in each replicate on slaughtering day; subsequently, the cecal bacterial load was determined as described by (27, 28).

### 2.8 Data statistical analysis

The collected data were analyzed by using General Linear Models (GLM), one-way analysis (SPSS software, 2019). Significant variations across treatment means were assessed utilizing Duncan’s multiple range test (29); moreover, statistical significance was declared at *p* < 0.05. Orthogonal polynomial contrasts were utilized to assess the linear and quadratic impacts of OLP. Graphs were produced utilizing GraphPad Prism version 8.0. In addition, the normality of the data distribution was assessed using the Shapiro–Wilk test, and the homogeneity of variances was evaluated using Levene’s test. All data met these assumptions; hence, no transformations were required. The following statistical model was employed:

$$Y_{\mathrm{ij}}=\mu +T_{i}+e_{\mathrm{ij}}$$

Where: *Y_ij_* = the *i*th observation; *μ* = the overall means; *T_i_* = effect of OLP treatments; *e_ij_* = the random experimental error.

## 3 Results

### 3.1 Growth efficiency

The outcomes in Table 3 illustrated that OLP addition in quail diets substantially (*p* < 0.05) improved LBW, BWG, and FI compared to the control. The inclusion of OLP at the 6% diet level yielded the best results of LBW (224.65 g), BWG (5.10 g), and FI (15.78) compared to other levels of OLP and those of the control. For FCR, no significant differences were detected among treatment groups for the overall experimental period (*p* > 0.05), although numerically lower (improved) FCR values were observed in the 4, 5, and 6% OLP groups compared to the control.

**Table 3:** Illustrate the impact of diet supplementation with OLP on quails’ growth performance.

Items	Treatments					SEM	*p*-value		
	Control	OLP 3%	OLP 4%	OLP 5%	OLP 6%		T	L	Q
LBW (g)									
Initial	10.48	10.41	10.48	10.43	10.50	0.03	0.956	0.848	0.677
LBW 2 W	37.86^b^	37.53^b^	51.35^a^	45.27^a^	47.28^a^	1.61	0.001	0.001	0.057
LBW 4 W	107.24^b^	108.98^ab^	109.85^ab^	131.51^a^	124.90^ab^	3.71	0.110	0.025	0.878
LBW 6 W	189.86^c^	197.82^bc^	207.84^b^	201.13^bc^	224.65^a^	3.59	0.004	0.001	0.427
BWG (g)									
BWG 1–15 d	1.95^b^	1.93^b^	2.92^a^	2.49^a^	2.62^a^	0.11	0.002	0.153	0.437
BWG 16–28 d	4.95^ab^	5.10^ab^	4.17^b^	6.16^a^	5.54^ab^	0.24	0.102	0.153	0.437
BWG 29–42 d	5.90^ab^	6.34^ab^	7.00^b^	4.97^b^	7.12^a^	0.29	0.101	0.548	0.725
BWG 1–42 d	4.27^c^	4.46^bc^	4.70^b^	4.54^bc^	5.10^a^	0.08	0.004	0.001	0.431
FI (g)									
FI 1–15 d	7.99^bc^	6.77^d^	10.80^a^	8.67^b^	7.20^cd^	0.39	0.000	0.766	0.000
FI 16–28 d	16.09	18.33	13.03	16.38	19.44	1.29	0.644	0.643	0.390
FI 29–42 d	19.63^ab^	22.07^a^	18.80^ab^	16.03^b^	20.70^ab^	0.77	0.122	0.421	0.390
FI 1–42 d	14.56	15.72	14.21	13.69	15.78	0.56	0.768	0.929	0.593
FCR (g/g)									
FCR 1–15 d	4.10^a^	3.54^a^	3.71^a^	3.51^a^	2.74^b^	0.56	0.022	0.003	0.376
FCR 16–28 d	3.19	3.56	3.01	2.74	3.52	0.20	0.730	0.913	0.560
FCR 29–42 d	3.44	3.50	2.85	3.27	2.91	0.18	0.782	0.390	0.893
FCR 1–42 d	3.42	3.52	3.02	3.04	3.09	0.13	0.726	0.295	0.738

^a,b,c,d,e^Means within the same row with different superscripts are significantly different (*p* ≤ 0.05).

OLP, olive leaf powder; LBW, live body weight; BWG, body weight gain; FI, feed intake; FCR, feed conversion ratio; SEM, standard error of the mean; T, treatment; L, linear; Q, quadratic.

### 3.2 Carcass characteristics

Table 4 showed that varying levels of OLP incorporation significantly (*p* < 0.05) influenced most carcass features. As shown, quails of the OLP 6 group yielded the highest carcass%, heart%, and liver% in comparison to other groups. On the other hand, the control group’s quails displayed the highest spleen percentage.

**Table 4:** Impact of diet supplementation with OLP on quails’ carcass traits.

Items	Treatments					SEM	*p*-value		
	Control	OLP 3%	OLP 4%	OLP 5%	OLP 6%		T	L	Q
Pre SW (g)	193.33^d^	212.33^bc^	206.66^c^	213.33^b^	221.66^a^	2.61	0.000	0.000	0.240
Carcass %	68.64^d^	68.91^cd^	72.06^ab^	72.79^a^	70.46^bc^	0.48	0.001	0.001	0.003
Liver %	3.44^a^	3.09^a^	2.61^b^	3.30^a^	3.35^a^	0.09	0.013	0.959	0.004
Heart %	0.75^c^	0.81^b^	0.89^a^	0.81^b^	0.88^a^	0.01	0.000	0.000	0.034
Gizzard %	2.04	2.11	1.99	2.08	1.97	0.03	0.808	0.569	0.681
T. edible %	5.48^a^	5.27^a^	4.72^b^	5.41^a^	5.44^a^	0.09	0.038	0.892	0.016
Spleen %	0.23^a^	0.11^bc^	0.10^c^	0.22^ab^	0.10^c^	0.02	0.038	0.183	0.367
Bursa %	0.12	0.09	0.09	0.12	0.09	0.01	0.774	0.722	0.699

^a,b,c,d,e^Means within the same row with different superscripts are significantly different (*p* ≤ 0.05).

OLP, olive leaf powder; Pre-SW, preslaughter; T. edible, total edible part; SEM, standard error of the mean; T, treatment; L, linear; Q, quadratic.

### 3.3 Blood indicators

The data presented in Table 5 illustrate a significant difference (*p* < 0.05) between treatments that were detected in most protein fractions, with the highest levels of TP and GLO recorded in the control group compared to the other OLP groups. Additionally, the results show lower levels of the enzymes ALT and AST in OLP groups 5 and 6, respectively, compared to the other treatments. The introduction of OLP into the quail diets led to improvements in their lipid profile, particularly in the OLP group 6, which exhibited the lowest levels of TG, TC, VLDL, and LDL, along with the highest levels of HDL compared to the control group.

**Table 5:** Effect of diet supplementation with OLP on quails’ blood biochemicals.

Items	Treatments					SEM	*p*-value		
	Control	OLP 3%	OLP 4%	OLP 5%	OLP 6%		T	L	Q
TC	261.00^a^	182.36^c^	233.41^b^	204.59^c^	192.24^c^	8.24	0.000	0.001	0.101
TG	345.46^a^	254.00^b^	205.74^bc^	253.36^b^	161.54^c^	19.30	0.008	0.002	0.370
HDL (mg/dL)	26.95^c^	57.55^a^	37.94^b^	40.64^b^	53.05^a^	3.11	0.000	0.003	0.208
LDL (mg/dL)	181.62^b^	74.01^c^	220.98^a^	152.28^ab^	101.22^bc^	18.02	0.000	0.040	0.033
VLDL (mg/dL)	69.09^a^	50.80^ab^	41.15^b^	69.34^a^	34.31^b^	4.45	0.006	0.0.024	0.852
CRT (g/dL)	0.75^a^	0.46^c^	0.72^ab^	0.55^bc^	0.46^c^	0.03	0.008	0.019	0.861
Urea (g/dL)	3.81^bc^	4.62^bc^	11.13^a^	7.01^b^	1.87^c^	0.94	0.001	0.677	0.000
TP (g/dL)	7.16^a^	7.08^a^	4.54^bc^	5.76^ab^	3.93^c^	0.39	0.002	0.000	0.886
Alb (g/dL)	2.46^b^	3.29^a^	2.37^b^	2.62^b^	2.10^b^	0.12	0.004	0.021	0.029
Glo (g/dL)	4.80^a^	3.91^ab^	2.28^bc^	3.24^abc^	1.94^c^	0.33	0.014	0.003	0.369
A/G ratio	0.65^b^	0.95^ab^	1.15^ab^	0.97^ab^	1.36^a^	0.09	0.157	0.031	0.800
AST (U/L)	184.16^a^	144.82^b^	182.65^a^	145.29^b^	117.43^c^	7.33	0.000	0.000	0.088
ALT(U/L)	23.44^a^	8.44^c^	16.60^b^	15.75^b^	12.85^bc^	1.44	0.001	0.018	0.026

^a,b,c,d,e^Means within the same row with different superscripts are significantly different (*p* ≤ 0.05).

OLP, olive leaf powder; TC, total cholesterol; TG, triglyceride; ALT, alanine transaminase; AST, aspartate aminotransferase; CRT, creatinine; TP, total protein; Alb, albumin; Glo, globulin; HDL, high-density lipoprotein; LDL, low-density lipoprotein; SEM, standard error of the mean; T, treatment; L, linear; Q, quadratic.

Table 6 shows the effects of OLP on the antioxidant activity of Japanese quail. All parameters were significantly affected (*p* < 0.05) by the OLP addition. It is obvious that the OLP groups, especially OLP6%, yielded the highest levels of GSH, GPx, SOD, and CAT compared to the untreated groups.

**Table 6:** Impact of diet supplementation with OLP on quails’ antioxidant enzymes.

Items	Treatments					SEM	*p*-value		
	Control	OLP 3%	OLP 4%	OLP 5%	OLP 6%		T	L	Q
GSH (ng/mL)	0.14^c^	0.22^bc^	0.35^a^	0.26^b^	0.42^a^	0.02	0.000	0.000	0.611
GPx (ng/mL)	0.13^c^	0.27^b^	0.27^b^	0.24^b^	0.35^a^	0.02	0.000	0.000	0.247
SOD (U/mL)	0.18^c^	0.22^c^	0.34^b^	0.24^c^	0.43^a^	0.01	0.000	0.000	0.030
CAT (mg/dL)	0.10^d^	0.18^c^	0.30^a^	0.24^b^	0.35^a^	0.02	0.000	0.000	0.513

^a,b,c,d,e^Means within the same row with different superscripts are significantly different (*p* ≤ 0.05).

OLP, olive leaf powder; GSH, Glutathione reduced; GP_X_, Glutathione peroxidase; SOD, superoxide dismutase; CAT, catalase; SEM, standard error of the mean; T, treatment; L, linear; Q, quadratic.

### 3.4 Meat quality

Table 7 shows the effect of adding different levels of OLP to quail diets on meat quality characteristics. All meat quality characteristics improved (*p* < 0.05) with the use of OLP. Compared to the control group and other treatments, the diet containing 6% extract achieved the best pH value. Furthermore, this diet exhibited the lowest values for brightness (L*) and yellowness (b*) and the highest values for redness (a*). Additionally, the diet containing 6% OLP demonstrated the highest water retention capacity and the lowest water loss during cooking.

**Table 7:** The effect of diet supplementation with OLP on quails’ meat quality traits.

Items	Treatments					SEM	*p*-value		
	Control	OLP 3%	OLP 4%	OLP 5%	OLP 6%		T	L	Q
Cooking loss %	19.95^a^	19.24^b^	19.00^c^	17.62^d^	17.52^d^	0.25	0.000	0.000	0.713
WHC	24.36^e^	24.92^d^	25.74^b^	25.48^c^	26.74^a^	0.21	0.000	0.000	0.000
PH	7.35^a^	7.00^b^	6.94^c^	6.76^d^	6.72^e^	0.05	0.000	0.000	0.000
Proteolytic	24.16^a^	20.76^ab^	19.06^ab^	20.08^ab^	15.66^b^	1.04	0.117	0.018	0.928
Lightness (L*)	23.88^e^	32.61^c^	38.91^b^	40.79^a^	30.80^d^	1.61	0.000	0.000	0.000
Redness (a*)	6.64^ab^	7.24^a^	7.34^a^	6.19^b^	5.04^c^	0.24	0.000	0.000	0.000
Yellowness (b*)	6.63^ab^	6.64^a^	6.62^b^	6.17^c^	5.09^d^	0.16	0.000	0.000	0.000

^a,b,c,d,e^Means within the same row with different superscripts are significantly different (*p* ≤ 0.05).

OLP, olive leaf powder; WHC, water-holding capacity; SEM, standard error of the mean; T, treatment; L, linear; Q, quadratic.

### 3.5 Microbiological examination of the cecum

**Figure 1:**
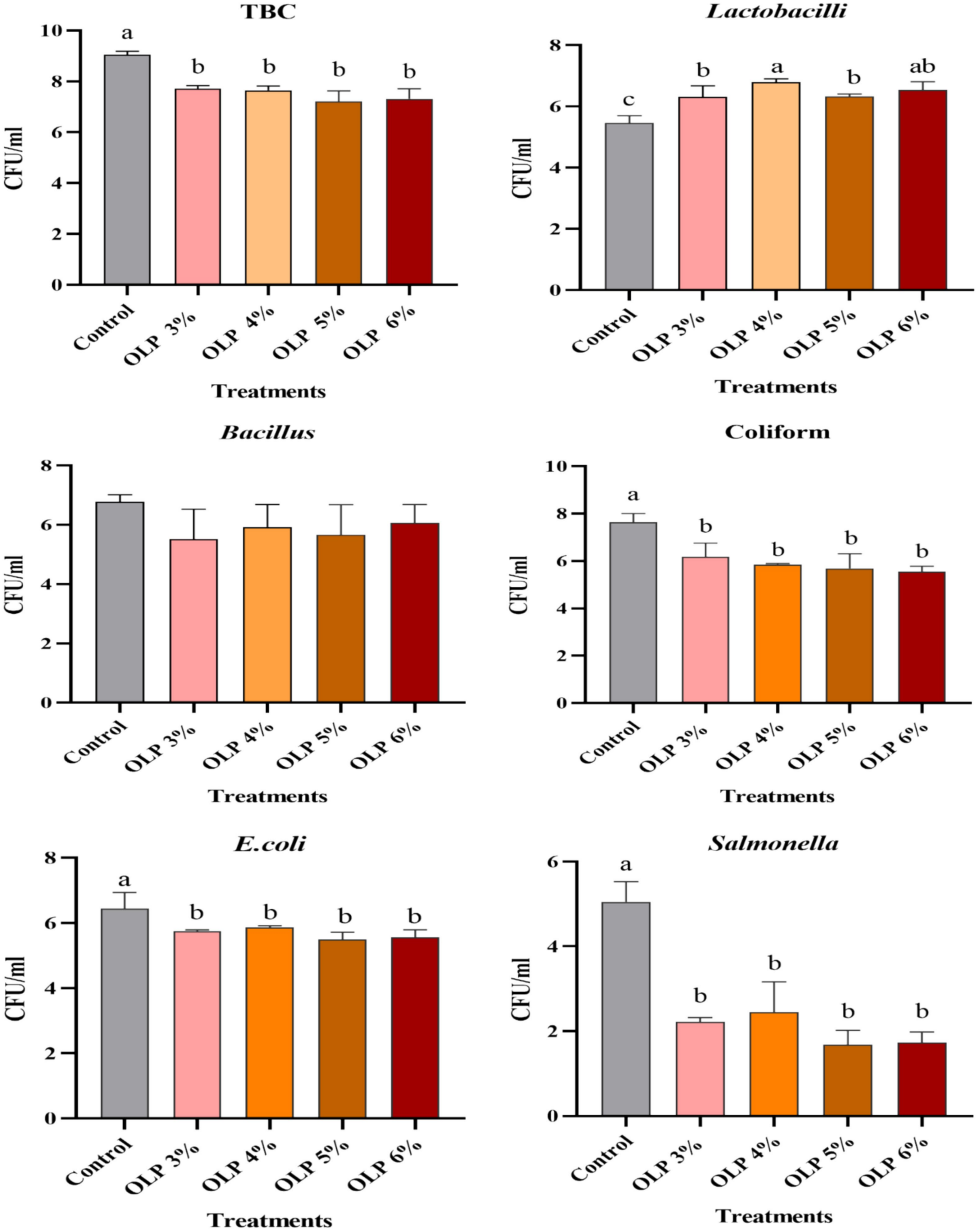
Effect of diet supplementation with OLP on quails’ cecal microbiota (Log^10^CFU/ml). ^a,b^Means within the same row with different superscripts are significantly different (*p* ≤ 0. 05). OLP, olive leaf powder; TBC, total bacterial count; CFU, colony-forming units.

The OLP treatment substantially influenced (*p* < 0.05) all caecal microbiota, except the Bacillus count, as illustrated in Figure 1. The OLP groups had the lowest levels of TBC, coliforms, *E. coli*, and *Salmonella* in comparison to the control. While OLP levels exerted marked effects (*p* < 0.05) on *Lactobacillus* counts, OLP treatments elevated these counts, particularly at concentrations of 4 and 6%, which exhibited the highest numbers.

## 4 Discussion

The olive leaf is a key component in phytotherapy, with Bucciantini et al. (30) indicating phenolic compounds from olive oil and leaves as potentially beneficial to health, especially with prolonged consumption. The results found that supplementing quail diets with 6% OLP significantly enhanced LBW, BWG, and FI. However, the magnitude of improvement in BWG was relatively small, suggesting limited biological relevance despite statistical significance. This modest effect may be because the basal diet already met or exceeded NRC (21) recommendations, leaving limited room for further growth enhancement. While FCR improvements were not statistically significant during the overall experimental period (*p* > 0.05), numerically lower FCR values were observed in the 4, 5, and 6% OLP groups compared to the control. The lack of statistical significance may be attributed to the relatively short 6-week experimental duration, the possible satiety effect of dietary fiber in OLP, or the adequacy of the basal diet. These outcomes are consistent with the results of El-Kelawy and Refaie (31), who found marked enhancements in quails’ growth efficiency, LBW, FI, FCR, and carcass traits with the inclusion of olive pomace in their diets at concentrations of 5 or 10%. Likewise, Adel et al. (32), who assessed the inclusion of OLE at variance levels (0.015, 0.03, and 0.06%) for growing quails’ diet, noted a notable improvement in previously estimated traits.

This boost can be ascribed to the multiple active compounds involved in OLE, including oleuropein and its derivatives, hydroxytyrosol, vanillic acid, caffeic acid, rutin, and vanillin, which may confer specified health benefits (33). Parallel results were recorded in broilers of the Sasso breed by Nassar et al. (34), who found that the best values of FCR were obtained from birds fed an OLP-supplemented diet at a level of 2%. OLE’s antimicrobial features make it a safe and sustainable growth promoter for poultry (35, 36). Nevertheless, the lack of significant improvement in FCR suggests that the bioactive compounds in OLP may exert their effects primarily through physiological and health-related pathways, rather than through a direct enhancement of dietary efficiency. This aligns with recent evidence suggesting that phytogenic additives function predominantly as modulators of oxidative status and gut health rather than direct growth promoters (37). Consequently, such mechanisms may yield moderate performance responses while profoundly influencing health-related parameters. This suggests that the observed improvements in LBW and BWG are likely mediated by enhanced intestinal health and antioxidant status rather than a substantial increase in nutrient absorption. Further research utilizing direct digestibility coefficients is warranted to validate these impacts. Moreover, OLE has been shown to positively alter the gut microbiota in poultry, further supporting its role as a functional feed additive.

The findings showed that varying concentrations of OLP incorporation significantly affected carcass features. Quails in the OLP 6 group exhibited the highest percentages of carcass, heart, and liver, while the control had the highest spleen. The findings align with Erener et al. (15), who noted that administering 0.6 g of olive leaf extract/kg of feed resulted in carcass weight and dressing in broiler chickens. The percentage of abdominal fat was decreased in untreated birds relative to all animals administered olive leaf extract. These findings imply that OLP may influence carcass improvement through metabolic modulation rather than substantial growth enhancement, consistent with Richards (38), who observed that FI is a pivotal determinant affecting animal growth rate and body composition during their lifespan. Almuhayawi et al. (37) noted that broiler chicks administered OLP exhibited decreased abdominal fat formation and a marked rise in net carcass weight and breast muscle mass. Moreover, Vasilopoulou et al. (39) indicated that OLP, especially at elevated doses, can enhance intestinal growth, as demonstrated by the augmented ratio of intestinal weight to carcass weight.

The outcomes are probably attributable to bioactive polyphenolic compounds and flavonoids prevalent in olive leaves, which demonstrate dose-dependent antioxidant properties, fostering cardiovascular and hepatic protection, thus augmenting organ growth while improving overall carcass productivity in Japanese quail (40). Moreover, Noreen et al. (41) indicated that such effects are increasingly linked to reduced systemic inflammation and oxidative burden, which may lower immune-related energy expenditure and redirect nutrients toward productive tissues. The enlarged spleen size in the control group aligns with recent research demonstrating that unfortified diets lead to larger lymphoid organs due to chronic oxidative stress or subclinical inflammation, contrasting with the immunomodulatory benefits of optimal oleuropein levels, which normalize spleen size without compromising immunity (37). This trend shows that OLP is a beneficial long-term plant supplement for quail nutrition. It improves carcass characteristics at moderate to high inclusion rates (2–6%) and reduces organ hypertrophy that is linked to base diets (42).

The findings indicate a notable difference in protein fractions across treatments, with the control group having higher TP and GLO levels. This reduction in TP and GLO in OLP-supplemented groups may reflect a shift in protein metabolism or reduced inflammatory stimulation, rather than a negative physiological impact, as globulins are often elevated under immune or stress conditions (37, 41). Moreover, the ALT and AST enzyme levels were lower in OLP groups 5 and 6;this decline strongly suggests a hepatoprotective effect of OLP, indicating improved liver integrity and reduced cellular leakage of transaminases, which are key biomarkers of hepatic stress (43, 44). Incorporating OLP into quail diets improved the lipid profile, particularly in group 6, which displayed the lowest TG, TC, VLDL, and LDL, alongside the highest HDL levels compared to the control. Such hypolipidemic effects are biologically relevant, as they indicate improved lipid metabolism and reduced risk of fat deposition, which aligns with enhanced carcass traits observed in this study (37, 45). Additionally, all indicators of antioxidants were substantially impacted by OLP addition, with OLP groups exhibiting the lowest GSH, GPx, SOD, and CAT levels, particularly the OLP 3%, which had the lowest values among all groups. However, this statement requires clarification, as the data (Table 6) actually demonstrate a significant increase in antioxidant enzyme activities with increasing OLP levels, particularly at 4 and 6%, indicating enhanced oxidative defense capacity rather than suppression (10, 46). Our results are consistent with the results of Bahshwan (14), which indicate that the dietary addition of OLE markedly improves lipid profiles and liver function in quails. A diet containing 0.03% OLE resulted in significant reductions in TC, VLDL, TG, and LDL, while increasing HDL. Additionally, OLE levels of 0.01, 0.02, and 0.03% contributed to notable improvements in ALT and AST. Furthermore, Nassar et al. (34) showed a significant increase in TP and GLO, whereas the A/G ratio declined in Sasso broilers fed a diet with 2% OLP compared to the control. This discrepancy with the present study may be attributed to differences in inclusion level, species-specific responses, or the bioavailability of phenolic compounds in powdered versus extracted forms (33, 37). Moreover, Nafea and Hussein (47) noted a substantial increase in blood TP and GLO levels in broilers with 2% olive leaves, alongside a decrease in TG and TC. Sarica and Toptas (48) reported a reduction in blood TC and LDL after a dietary addition of 0.2 g OLP/kg of quail feed. Almuhayawi et al. (37) found that the injection of OLP enhanced the lipid profiles and ALT or AST in broilers compared to the control.

Agah et al. (43) identified a reduction in TC, TG, ALT, and AST levels in broilers fed diets with 0.2 or 0.4 g OLP/kg diet. Parsaei et al. (49) observed a significant decrease in blood TC, HDL, LDL, and VLDL levels in broilers supplemented with an OL-diet at levels of 0.5 and 0.75%, relative to the untreated group. However, unlike some previous studies (50), which reported that OLE insignificantly influenced serum TC, LDL, and HDL, our study found significant lipid-lowering effects. This inconsistency highlights the importance of dose-dependent responses and suggests that higher inclusion levels (e.g., 6% OLP) may be required to elicit measurable metabolic changes in quail (37, 50). Also, this discrepancy may be explained by differences in dosage (6% OLP vs. lower levels), bird species (quail vs. broilers), or the phenolic profile of the olive leaf powder used. Variability in oleuropein and hydroxytyrosol content due to harvest time, drying method, and storage conditions may also contribute. The hepatoprotective and hypolipidemic effects observed in the present study may be mechanistically linked to the ability of olive polyphenols to modulate lipid metabolism and reduce oxidative stress via redox-sensitive signaling pathways such as Nrf2 Soleimani et al. (44). Activation of Nrf2 signaling enhances the transcription of antioxidant and detoxifying enzymes, thereby improving hepatic resilience and systemic metabolic homeostasis (44, 51). Compounds found in OLP may possess the capacity to suppress the release of inflammatory mediators and cytokines, consequently reducing inflammation in the kidneys and liver (52). Moreover, the antioxidants present in OLP can safeguard these organs from damage linked to oxidative stress; the application of OLP can maintain the normal functionality of renal and hepatic cells by diminishing oxidative stress and inflammation. This unified antioxidant and anti-inflammatory mechanism offers a clear rationale for the concurrent enhancement of liver enzymes and lipid profile (53, 54). Chemicals in olive leaf (OL) may enhance liver metabolism of harmful substances, reducing kidney workload (53). Oleuropein, a significant antioxidant polyphenol in OL, reduces lipid peroxidation and cholesterol levels by neutralizing free radicals (54). OL may lower dietary cholesterol absorption in birds by binding cholesterol and promoting bile acid metabolism, with polyphenols increasing bile acid production, thereby aiding in cholesterol elimination (45). Collectively, these mechanisms indicate that OLP acts not merely as a dietary additive but as a metabolic modulator influencing hepatic function, lipid turnover, and systemic oxidative balance (10, 37, 44).

Among the key antioxidant enzymes are reduced GSH and SOD, with MDA serving as a biomarker for lipid damage due to oxidative stress (51). The study indicates that OLP treatments significantly enhance the quail’s antioxidant status, aligning with findings by Jemai et al. (46) that hens given OLE show increased SOD levels and reduced MDA concentrations. This enhancement is strongly supported by recent studies demonstrating that olive leaf polyphenols upregulate endogenous antioxidant enzymes through transcriptional activation of antioxidant defense systems (10). OLP may strengthen the body’s defense against oxidative stress, supporting the production of antioxidant enzymes and stabilizing cell membranes, thus lowering MDA levels (55). Also, a recent study by Al-Shammari et al. (56) demonstrated that compounds like oleuropein and hydroxytyrosol neutralize reactive oxygen species, mitigating oxidative damage. Additionally, research shows increased T-SOD and GPx levels in broilers consuming diets with enzymatically fermented OP, attributed to its phenolic components (57). These improvements emphasize a stronger impact of OLP on metabolic health and oxidative status than on growth performance.

OLP usage significantly enhanced all meat quality characteristics. The diet containing 6% extract yielded the best pH, the lowest L* and b*, and the highest a*. It also exhibited superior water retention capacity and minimized water loss during cooking. Moreover, the findings align with Xie et al. (50) and Vasilopoulou et al. (39), indicating that the incorporation of 0.3% OLE into the diet diminished breast meat shear force, elevated GSH levels, enhanced T-SOD and GSH-Px activities, and decreased MDA levels. This indicates that lipid peroxidation and protein denaturation were diminished, resulting in enhanced softness, color fidelity, and moisture retention. Likewise, 1% oleuropein-enriched OLE improved the color of thigh meat, increased tenderness, and maintained intestinal health without hindering growth, supporting the dose-dependent effectiveness of polyphenols in regulating postmortem glycolysis for ideal pH reduction and myoglobin preservation against metmyoglobin development, thereby enhancing sustainable meat quality through natural feed additives (58, 59). The polyphenols found in OLP can reduce the drop in muscle pH after slaughter by regulating microbial growth and metabolic processes, often leading to a more stable final meat pH and slowing down spoilage (60). In addition, these improvements in meat quality when OLP or extract is added to poultry feed are due to biochemical mechanisms, as the polyphenolic antioxidants found in olive leave help reduce the oxidation of fats and proteins, stabilize meat quality after slaughter, and improve water retention, leading to improved muscle condition and reduced losses during cooking.

The OLP groups showed the lowest concentrations of TBC, coliforms, *E. coli*, and *Salmonella* compared to the control. OLP treatments significantly increased *Lactobacillus* counts, especially at 4 and 6%, which were the highest. This shift in gut microbiota indicates that OLP may function as a natural antimicrobial and prebiotic agent, contributing to improved intestinal health rather than direct growth enhancement. However, OLP supplementation did not significantly affect the Bacillus count, indicating that not all beneficial bacterial genera respond equally to olive leaf polyphenols. These outcomes align with the results Adel et al. (32) demonstrated that 0.03 and 0.06% OLP enhance the proliferation of *Lactobacilli* and diminish the prevalence of pathogenic bacterial species in the ceca of growing quail. OLP contains many bioactive chemicals with strong antibacterial properties that can compromise bacterial cell membranes, resulting in cell death and the release of internal components or impair their enzymatic systems (61, 62). Additionally, several bioactive chemicals included in OL may impede bacterial communication, or quorum sensing, hence decreasing the capacity of pathogens to develop biofilms and propagate illnesses (63). Also, Plamada and Vodnar (64) indicated that the fiber and polyphenol content in olive oil functions as prebiotics, promoting the multiplication of beneficial bacteria such as *Lactobacillus* and *Bifidobacterium*. Probiotic-enhanced fermentation improves the production of SCFA, such as butyrate, which fortifies intestinal cells and suppresses pathogen proliferation (65).

## 5 Conclusion

In the context of the current 6-week experiment, the integration of OLP into the quails’ diet yielded significant advantages for the Japanese quail growth, performance, and production. The results revealed an increase in growth performance, improvements in carcass attributes, enhancements in hematological parameters, a further boost in antioxidant activity, and a superior composition of intestinal microbiota. However, the improvements in growth performance were modest and should be interpreted with caution in terms of biological relevance. Among the investigated inclusion levels, 6% OLP proved most favorable in improving growth performance, lipid profile, antioxidant enzyme activity, and meat quality traits, but both 4 and 6% OLP comparably increased the cecum Lactobacillus population. Accordingly, inclusion of 6% OLP is advised as the ideal dietary intake based on the parameters of the current study. The results of this study indicate that OLP demonstrates potential as a sustainable and safe feed supplement for Japanese quail production. Overall, OLP appears to act primarily as a functional feed additive that supports physiological health, antioxidant status, and microbial balance rather than a strong growth promoter. Further investigation is necessary to discover the biological mechanisms of the bioactive compounds in OLP, estimate their long-term impacts on quail products, and ascertain their economic feasibility in commercial quail farming enterprises.

## Data Availability

The raw data supporting the conclusions of this article will be made available by the authors, without undue reservation.
